# COGNITIVE DECLINE AND FALLS ARE INDEPENDENT RISK FACTORS FOR SWALLOWING DYSFUNCTION IN OLDER PEOPLE

**DOI:** 10.1093/geroni/igad104.3232

**Published:** 2023-12-21

**Authors:** I-Ting Liu

**Affiliations:** E Da Hospital, Kaohsiung City, Kaohsiung, Taiwan (Republic of China)

## Abstract

Aging, oropharyngeal lesion, neuromuscular and progressive neurological disease such as dementia are well-known risk factors of dysphagia. The aim of this study is to evaluate the significant risk factors of swallowing dysfunction in clinical practice. 100 subjects aged 65 or older who received Comprehensive Geriatric Assessment (CGA) were enrolled at EDA Hospital, Taiwan, form January 2022 to February 2023 (IRB: EMRP-110-108). Exclusion criteria: unable to communicate 、advanced disease or totally dependent. CGA was performed by well-trained nurses and geriatrician. Swallowing dysfunction was identified by abnormal swallow exam or related complaints. Cognitive impairment was defined as Short Portable Mental State Questionnaire (SPMSQ) < 8 (SPMSQ < 6 in those without education) or with diagnosed dementia. Binary logistic regression was constructed to assess the independent associated factors of dysphagia. Basic demographic analysis showed that patients with dysphagia were older, had poor ADL, higher number of geriatric syndromes and chronic comorbidities. Multivariate analysis revealed that cognitive decline (OR: 82.74, p=0.035) and falls (OR: 45.17, p=0.011) are still independent associated factors of swallow dysfunction after adjustment for age, sex, BMI, socioeconomics, other geriatric syndromes, chronic comorbidities including stoke and Parkinson disease. Dementia was known as one of the risk factors of dysphagia. Another small follow-up study demonstrated that falls risk is predictive of dysphagia in Parkinson’s disease. Through this study, participants will be able to identify the high-risk group of dysphagia. Prevention such as pneumococcus vaccination, oral hygiene, and speech therapy can be applied on those who have a fall history and clinical cognitive impairment.

